# On endocrine disruption at the workplace – how to get from suggestive to conclusive evidence?

**DOI:** 10.5271/sjweh.3897

**Published:** 2020-07-01

**Authors:** Dr Jens Peter Bonde

**Affiliations:** Department of Occupational and Environmental Medicine, Bispebjerg and Frederiksberg Hospital, University of Copenhagen, Copenhagen, Denmark [e-mail: Jens.Peter.Ellekilde.Bonde@regionh.dk]

Unusual clusters and outbreaks of reproductive disorders during past decades remind us of the devastating health consequences and human suffering to which exposure to industrial chemicals and pharmaceuticals may give rise. Birth defects caused by gestational exposure to thalidomide, vaginal cancer by diethylstilbestrol, azoospermia by dibromochloropropane, cerebral palsy by methyl mercury, and fetal wasting syndrome by chlorinated biphenyls are all inscribed in medical history as tragic mementos of ignorance and neglect in the past (1).

In parallel with the fast-increasing entrance of women into the workforce, interest in occupational reproductive hazards accelerated in the Nordic countries in the 1970s and 80s. Women working in healthcare, industry, and agriculture are potentially exposed to high levels of chemicals, resulting in possibly substantial risk of toxicity to their unborn child.

Systematic epidemiologic research addressing reproductive hazards at the workplace has fostered thousands of papers from the 1980s to present day. Reports of associations between a large range of exposures and reproductive outcomes spanning infertility, adverse pregnancy outcomes, and childhood cancer and cognitive impairment are abundant. Many studies suggest that in particular organic solvents, pesticides and heavy metals are occupational reproductive hazards, but – with noteworthy exceptions – few specific exposures survive critical systematic review to become established reproductive toxicants in the workplace (2).

The introduction of the endocrine disruption hypothesis more than 20 years ago has dramatically renewed interest in reproductive toxicity and provided a new framework for research with its focus on a genuine mechanism for toxic effects related to disruption of endogenous hormone homeostasis. Initially it was thought that toxicants interfere with fetal development by mimicking or enhancing effects of natural estradiol (3), but, as evidence accumulated, the hypothesis became much broader. A capacious amount of experimental studies have demonstrated that adverse reproductive outcomes may be related to endocrine disruption of fetal development through xenobiotic interaction with several types of hormone receptors or through interference with synthesis, secretion, transport, metabolism, or degradation of natural hormones (4). If this results in pathologic outcomes, it is not always obvious, and the relevance for human exposure scenarios remain hypothetical (5).

Concern increased as it became evident that virtually all people across the globe host in their bodies not only biopersistent organic pollutants such as DDT and PCBs but also substances with shorter biological half-lives that do not accumulate, such as phthalates (4). The European Chemicals Agency defines endocrine disruptors as chemicals where at least one study has shown not necessarily pathologic or adverse endocrine effects in an intact organism (typically small rodents). The list of such chemicals has become steadily more voluminous and heterogenous and now embraces many of the compounds that have been extensively studied earlier without reference to endocrine disruption – such as organic solvents, cytotoxic drugs, many pesticides, metals, and polyaromatic hydrocarbons (6).

Already in 1999, an editorial advocated for the need to address reproductive toxicity of endocrine disrupting chemicals at the workplace (7), but – in contrast to many studies on exposures in the general environment – to date only few epidemiological studies explicitly addressing endocrine disruption at the workplace have been published (8–14). Therefore, the study by Shirangi and colleagues in this issue of *The Scandinavian Journal of Work, Environment and Health* is highly welcomed (15). They report associations between occupational exposure to endocrine disrupting chemicals during pregnancy and risk of impaired fetal growth in an English prospective birth cohort, including some 4000 pregnant women. A job exposure matrix (JEM) was used to assess exposure. Substantially elevated relative risks of 3–5 are reported for two of seven classes of endocrine disrupting chemicals – pesticides and phthalates – while no increased risk was found for organic solvents, metals and other exposures. Besides the prospective design, important strengths of this study include a high response and an impressive amount of detailed data on numerous potentially confounding factors, which are accounted for in analyses. The study is also innovative in its use of optimal birth weight in terms of the ratio of observed-to-predicted birth weight as an alternative measure of fetal growth (16). The predicted birth weight was derived from the birthweight distribution among singleton Caucasians without known pathological determinants, such as smoking and birth defects, and was standardized according to gestational age, fetal gender, maternal height, and parity (17). The optimal birth weight measure is less dependent on an appropriate reference population than the traditional small-for-gestational-age measure but neither accounts for ethnicity (almost 30% was of South Asian origin) nor solves the problem of potential collider bias, which is introduced by standardization on gestational age (18).

Although Shirangi et al’s study is supported by findings in few earlier studies (9,10), it is reasonable to ask whether we have reached a point where sufficient evidence has accumulated that allows us to provide specific recommendations to work and health authorities. One crucial issue is exposure assessment. Shirangi et al used a qualitative expert-rated JEM tabulating groups of jobs into unlikely, possible, or probable exposure to one or more of ten classes of endocrine disrupting chemicals. JEM may be powerful tools to obtain complete data on exposure without risk of recall bias and at low cost (19, 20) – in particular when a crude exposure assignment based on groups of job titles can be refined by access to individual data on industries and job tasks as in the Shirangi et al study. However, occupational exposure to some endocrine disrupting chemicals, such as phthalates and bisphenol A, has only been quantified by workplace and biological measurement to a very limited extent (21, 22). For example, hairdressers and cosmetologists are the most prevalent occupations exposed to phthalates according to the endocrine disruption JEM, but this finding has not been documented by ambient air, skin or biological samples showing that these occupations do confer an exposure exceeding the background exposure (23, 24). Since phthalate metabolites are detectable in urine in almost everybody, sources of exposure other than occupational are important. Measurements of occupational exposure and validation of the endocrine disruption JEM by quantitative data is therefore highly warranted. Other occupational exposures, such as pesticides, solvents and metals, are much better documented by workplace measurements, thus their respective JEM may be more valid – in particular when a JEM either provides estimates of exposure to specific compounds or classes of compounds that share mechanism of action, toxic potency and kinetics. The above definition of an endocrine disruptor is far too broad to fulfil this criterion. We need exposure–response data to evaluate causality and provide a basis for regulation and prevention. To obtain exposure–response data, we probably have to get information on specific compounds or group of compounds known to operate by similar mechanism. At present we do not know if current occupational threshold limits for organic solvents, metals and pesticides need to be updated with data on endocrine activity and whether some exposure time windows are more sensitive than others.

Numerous large birth cohorts have been established in Europe over the past 20 years (25), and many of these are designed to examine effects of environmental and occupational exposures (26, 27). It has become feasible to include a whole range of new outcomes that perhaps are more sensitive to endocrine disruption by chemicals during fetal life compared to outcomes observed at birth – for instance premature or delayed puberty (28, 29), infertility in young people (30), and airway disorders (31)

The health consequences of reproductive hazards at the workplace may be grave for the individual and society. Ensuring a working environment that protects reproductive health must be a high priority. With reference to the precautionary principle, it may be tempting to exclude pregnant women from work that may carry the slightest theoretical risk. But this approach may have strong social and economic consequences for the women who thus do not necessarily benefit from the doubt. We need to know more. While experimental studies are important for risk assessment, we rely on epidemiology to address real world occupational exposure scenarios. Epidemiological methods and populations are available, but to ensure progress we need provision of resources to refine assessment of occupational exposure to endocrine disrupting chemicals by ambient air and biological measurements.

The author thanks Karin Sørig Hougaard, Sandra Tøttenborg, Vivi Schlünssen and Alex Burdorf for their thoughtful comments to the initial draft.
